# Distinct effects of folate pathway genes *MTHFR* and *MTHFD1L* on ruminative response style: a potential risk mechanism for depression

**DOI:** 10.1038/tp.2016.19

**Published:** 2016-03-01

**Authors:** N Eszlari, D Kovacs, P Petschner, D Pap, X Gonda, R Elliott, I M Anderson, J F W Deakin, G Bagdy, G Juhasz

**Affiliations:** 1 Department of Pharmacodynamics, Faculty of Pharmacy, Semmelweis University, Budapest, Hungary; 2 MTA-SE Neuropsychopharmacology and Neurochemistry Research Group, Hungarian Academy of Sciences, Semmelweis University, Budapest, Hungary; 3 Department of Clinical and Theoretical Mental Health, Kutvolgyi Clinical Center, Semmelweis University, Budapest, Hungary; 4 Neuroscience and Psychiatry Unit, School of Community Based Medicine, Faculty of Medical and Human Sciences, The University of Manchester, Manchester, UK; 5 Manchester Academic Health Sciences Centre, Manchester, UK; 6 Manchester Mental Health and Social Care Trust, Manchester, UK; 7 MTA-SE-NAP B Genetic Brain Imaging Migraine Research Group, Hungarian Academy of Sciences, Semmelweis University, Budapest, Hungary

## Abstract

Alterations in the folate pathway have been related to both major depression and cognitive inflexibility; however, they have not been investigated in the genetic background of ruminative response style, which is a form of perseverative cognition and a risk factor for depression. In the present study, we explored the association of rumination (measured by the Ruminative Responses Scale) with polymorphisms of two distinct folate pathway genes, *MTHFR* rs1801133 (C677T) and *MTHFD1L* rs11754661, in a combined European white sample from Budapest, Hungary (*n*=895) and Manchester, United Kingdom (*n*=1309). *Post hoc* analysis investigated whether the association could be replicated in each of the two samples, and the relationship between folate pathway genes, rumination, lifetime depression and Brief Symptom Inventory depression score. Despite its functional effect on folate metabolism, the *MTHFR* rs1801133 showed no effect on rumination. However, the A allele of *MTHFD1L* rs11754661 was significantly associated with greater rumination, and this effect was replicated in both the Budapest and Manchester samples. In addition, rumination completely mediated the effects of *MTHFD1L* rs11754661 on depression phenotypes. These findings suggest that the *MTHFD1L* gene, and thus the C_1_-THF synthase enzyme of the folate pathway localized in mitochondria, has an important effect on the pathophysiology of depression through rumination, and maybe via this cognitive intermediate phenotype on other mental and physical disorders. Further research should unravel whether the reversible metabolic effect of *MTHFD1L* is responsible for increased rumination or other long-term effects on brain development.

## Introduction

Major depressive disorder is an etiologically heterogeneous condition,^1^ in which a core and specific feature is depressive rumination.^2^

Ruminative response style, which is sometimes referred as depressive rumination, can be defined in several ways.^3^ In a broader sense, it is a form of cognitive inflexibility or perseverative cognition that prolongs the negative effect of everyday life stressors.^4, 5^ In addition, it may involve an impairment of the top-down cortical control on mnemonic processes, resulting in unwanted and uncontrollable dwelling on intrusive memories.^6^ Ruminative response style in association with depression is perceived as thinking repeatedly and passively about one's feelings and problems related to distress and depressed mood, thus exacerbating and prolonging depression.^7^ Indeed, it has been demonstrated that ruminative response style predicts the onset and level of future depression.^7, 8^ These facts suggest that ruminative response style or shortly rumination (as it is generally addressed) is a potential intermediate phenotype for depression.

Rumination is a moderately heritable trait with a 20–40% heritability rate based on twin studies.^9, 10^ Most importantly, phenotypic correlation between depressed mood and rumination appears to be explained mainly by shared genetic factors.^9, 10^ Thus, genetic risk factors for rumination are likely to share a pathophysiological role in the development of depression; however, hypothesis-free genome-wide association studies with rumination have not yet been reported. Using a candidate gene approach, both dopaminergic and serotonergic genes have been implicated in rumination (*DRD2* (ref. 11) *COMT* (ref. 12) and serotonin transporter *SLC6A4* (ref. 13)), and also extensively investigated in relation to cognitive flexibility and response inhibition (for review see Logue and Gould^14^). In addition, genes related to neuronal and synaptic plasticity (*KCNJ6* (ref. 15), *CREB1* (refs. 15, 16) and *BDNF* (refs. 13, 16) and stress response (*NR3C2* (ref. 17)) showed significant associations with rumination.

Altered folate function and the linked one-carbon cycle have long been implicated in the pathogenesis of depression and also in cognitive inflexibility or perseverative cognition, yet their possible role in rumination has not been investigated. More specifically, folate is necessary to the catabolism of homocysteine, and folate deficiency and elevated homocysteine are related to both depression and inflexible cognition.^18, 19, 20^ These metabolic changes are also associated with altered brain monoamine metabolism and impaired neuronal plasticity.^19, 20^

The most investigated genetic variant of the folate pathway is the *MTHFR* C677T (rs1801133) polymorphism, which leads to an alanine (C allele) to valine (T allele) substitution in the 5,10-methylenetetrahydrofolate reductase (MTHFR) protein. The *MTHFR* 677 T allele codes a thermolabile and less active enzyme, which is associated with decreased folate and increased homocysteine levels.^21^ Despite its strong metabolic impact, this polymorphism has shown conflicting results in genetic association studies of inflexible cognition,^19, 22^ major depression^1, 23^ and other neuropsychiatric disorders, such as Alzheimer's disease (AD),^24^ bipolar disorder and schizophrenia.^23^

Three studies reported that the A allele of a polymorphism (rs11754661) in another gene involved in folate metabolism, *MTHFD1L,* showed a genome-wide significant association with late-onset AD,^25, 26, 27^ although one study was negative.^28^ *MTHFD1L* encodes the human mitochondrial monofunctional 10-formyl-tetrahydrofolate synthetase (C_1_-THF synthase) enzyme.^29^ The A allele, similar to the *MTHFR* 677 T allele, is associated with increased homocysteine concentrations.^30^ In addition, this enzyme is obligatory for the production of mitochondrial formate, the essential substrate for cytoplasmic purine and thymidylate biosynthesis, methionine biosynthesis and amino-acid metabolism.^29, 31^ Although the direct link between rumination and AD is as yet only hypothetical,^32^ the association of the *MTHFD1L* gene with age-related cognitive decline, together with its pivotal role in normal neuronal development,^31, 33, 34^ suggests that it could be relevant in cognitive processes throughout the life.

In the present study, we investigated the association of ruminative response style with *MTHFR* rs1801133 and *MTHFD1L* rs11754661. We hypothesized that genetic variants in the folate pathway are associated with rumination, which is a cognitive risk factor for depression. In addition, we examined the relationship between folate pathway genes, rumination and depression phenotypes.

## Materials and methods

This study was part of the European Union-funded NewMood study (New Molecules in Mood Disorders, Sixth Framework Program of the EU, LSHM-CT-2004-503474), which was carried out in accordance with the Declaration of Helsinki and approved by local Ethics Committees (North Manchester Local Research Ethics Committee, Manchester, UK; Scientific and Research Ethics Committee of the Medical Research Council, Budapest, Hungary).

### Participants

Participants aged 18–60 years were recruited through general practices and advertisements from Budapest, Hungary, and through general practices, advertisements and a website from Greater Manchester, UK. All participants provided written informed consent. *N*=2204 subjects (*n*=895 from Budapest and *n*=1309 from Manchester) provided information about gender, age and rumination by filling out the NewMood questionnaire pack (in English or Hungarian, as appropriate)^16^ and were successfully genotyped for *MTHFR* rs1801133 by providing DNA with a genetic saliva sampling kit. *MTHFD1L* rs11754661 was successfully genotyped in 2120 subjects among those who provided information about gender, age and rumination (*n*=862 from Budapest and *n*=1258 from Manchester). All subjects were of European white ethnic origin, and had no relatives participating in the study.

### Phenotypic assessment

We used the 10-item Ruminative Responses Scale to measure rumination,^8^ and calculated rumination score as a continuous weighted score: the sum of item scores divided by the number of items completed. The NewMood questionnaire pack also included measures of two distinct depression phenotypes. Current depressive symptoms were measured by the depression items plus the additional items of the Brief Symptom Inventory (BSI),^35^ using a weighted score (see above, at rumination). Reported lifetime depression was derived from the background questionnaire and had been validated in a subpopulation with face-to-face diagnostic interviews.^16^

### Genotyping

For genotyping we collected buccal mucosa cells and extracted genomic DNA according to a validated method.^36^ The two single-nucleotide polymorphisms (SNPs), *MTHFR* rs1801133 and *MTHFD1L* rs11754661, were genotyped with the Sequenom MassARRAY technology (Sequenom, San Diego, CA, USA, www.sequenom.com). All laboratory work was blinded with regard to phenotype and performed under the ISO 9001:2000 quality-management requirements.

### Statistical analyses

PLINK v1.07 (http://pngu.mgh.harvard.edu/purcell/plink/) was used to calculate Hardy–Weinberg equilibrium for *MTHFR* rs1801133 and *MTHFD1L* rs11754661, and to build linear regression models for rumination score as an outcome variable. *MTHFR* rs1801133 or *MTHFD1L* rs11754661, respectively, and age, gender and population (Budapest or Manchester) were the predictor variables in all regression equations. With rs1801133, additive, dominant and recessive models were run in the combined sample. However, with rs11754661, we did not run the recessive model because of the low number of those homozygous for the minor allele. Bonferroni-corrected two-tailed *P*⩽0.010 was used as a significance threshold, and *P*⩽0.020 as a trend threshold, for the main analysis. As *post hoc* analysis, we investigated the significant effects separately in the Budapest and Manchester samples to test possible replications. In addition, we ran *post hoc* regression analyses similarly to the ones described above, for lifetime depression and current depression score. Furthermore, we tested the mediating role of depression phenotypes on rumination or the mediating role of rumination on depression phenotypes by including the mediating phenotype(s) as covariate(s) to test shared explained variance by the genes and these phenotypes. For *post hoc* statistical testing two-tailed *P*⩽0.05 threshold was used. We applied the parametric statistical methods based on the central limit theorem, as we have large samples (*n*>200).^37^ Descriptive statistics for the combined and separate samples were calculated with IBM SPSS 20.0 (IBM, Armonk, NY, USA) for Windows. We used Quanto for power calculations (http://biostats.usc.edu/Quanto.html), and OpenMeta[Analyst] for meta-analyses of genetic effects in the separate Budapest and Manchester samples (http://www.cebm.brown.edu/open_meta/download.html). To enhance the speed of the PLINK analysis, individually written R-scripts were used.^38^

## Results

The minor allele is T for *MTHFR* rs1801133 and A for *MTHFD1L* rs11754661. Both SNPs were in Hardy–Weinberg equilibrium in Budapest, Manchester and in the combined sample. For rs1801133, *P*-values are as follows: *P*=0.384 in Budapest, *P*=0.670 in Manchester and *P*=0.852 in the combined sample. For rs11754661, *P*=1 in Budapest, *P*=0.064 in Manchester and *P*=0.112 in the combined sample. Description of total sample and for Budapest and Manchester separately is given in Table 1. As we can see in Table 1, the Budapest and Manchester samples differ significantly in age, rs11754661 genotype frequencies, rumination and both depression phenotypes, which makes it reasonable to include population as a predictor variable in the regression equations.

**Figure 1:**
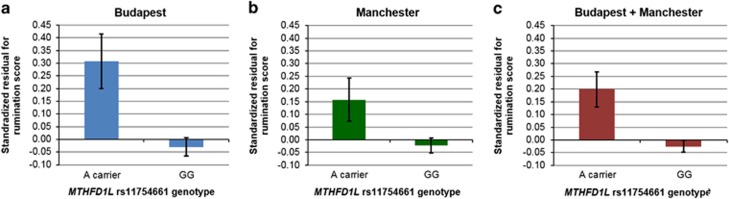
Means (and its s.e.'s) of standardized residuals for rumination score, according to the rs11754661 genotype. General linear models were created for rumination score as an outcome variable, separately in Budapest, Manchester (with age and gender as covariates) and in the combined sample (with age, gender and population as covariates). Standardized residuals of these models were then displayed according to the *MTHFD1L* rs11754661 genotype, thus representing the variance of rumination not accounted for by age, gender and population. A carriers show higher rumination than those with GG genotype in Budapest (**a**), Manchester (**b**) and also in the combined sample (**c**).

In our combined sample *MTHFR* rs1801133 did not show any significant effect on rumination (Table 2). Considering the effect of *MTHFD1L* rs11754661 on rumination, with age, gender and population as covariates in the regression equations, the A allele showed a significant positive association with rumination score in the combined sample, both in additive and dominant models (Table 2). These findings remained significant after Bonferroni correction for multiple testing. *Post hoc* analysis showed that the effect of the A allele remained statistically significant at a nominal (uncorrected) level in the Budapest and Manchester samples separately (Table 3). To visualize and meta-analyze these associations, standardized residuals were calculated for rumination score (separately in Budapest and Manchester and in the combined sample), by partialling out variance accounted for by age, gender and population (this latter only in the combined sample). The means (with s.e.'s) of these residuals are represented according to the rs11754661 genotype in Figure 1. A marked difference in rumination can be seen between A carriers and those with the GG genotype in Budapest (Figure 1a), Manchester (Figure 1b) and also in the combined sample (Figure 1c). We entered the means and s.d.'s of these standardized residuals of Budapest (0.31±0.930 in A carriers and −0.03±1.001 in the GG group) and Manchester (0.16±1.066 in A carriers and −0.02±0.988 in the GG group) into OpenMeta[Analyst] to calculate a combined mean difference between genotypes, in a continuous random-effects model. The combined mean difference and its s.e. is significant: 0.246±0.079 (*P*=0.002), underpinning our significant linear regression results in Budapest, Manchester and the combined sample (Tables 2 and 3). No significant between-study heterogeneity exists (tau^2^=0.002; *Q*=1.235; *P*=0.267; *I*^2^=19%), pointing out the validity of conducting a mega-analysis for the rs11754661 effect in the combined sample (Table 2).

Similarly, we ran a meta-analysis for the combined mean difference between *MTHFR* rs1801133 T carriers and those with CC genotype. The means (and their s.d.'s) of the standardized residual for rumination score are as follows: −0.04±0.993 in T carriers in Budapest, 0.05±1.006 in the CC group in Budapest; and −0.03±1.002 in T carriers in Manchester, 0.05±0.995 in the CC group in Manchester. A continuous random-effects model yielded a combined mean difference (and its s.e.) of −0.084±0.043 (*P*=0.051), underpinning the result of the dominant model among linear regressions in that T carriers nominally tend to ruminate less than the CC group (Table 2). As in the case of rs11754661, the unsignificant heterogeneity test results between Budapest and Manchester (tau^2^<0.001; *Q*=0.013; *P*=0.909; *I*^2^=0%) also validate mega-analysis of the rs1801133 effect on rumination in the combined sample (Table 2).

We ran *post hoc* analyses in the combined sample to unravel whether the association between rs11754661 and rumination is mediated by depression phenotypes. Rumination shows a significant positive association with both of our depression phenotypes: Pearson correlation coefficient *r*=0.581 (*N*=2117; *P*<0.001) for BSI depression score and *t*=−22.022 (*N*=2120; *P*<0.001) for lifetime depression (mean rumination score is 1.909±0.014 in those who did not and 2.429±0.019 in those who did report lifetime depression). The *MTHFD1L* rs11754661 A allele also associates positively (either significantly or as a trend) to both depression phenotypes. Namely, for BSI depression score, its *β*=0.098; *t*=1.695; *P*=0.090 in an additive, and *β*=0.118; *t*=1.897; *P*=0.058 in a dominant PLINK linear regression model. For lifetime depression, its odds ratio (OR)=1.354; *t*=2.173; *P*=0.030 in an additive, and OR=1.405; *t*=2.271; *P*=0.023 in a dominant PLINK logistic regression model. *N*=2117 in BSI depression and *N*=2120 in lifetime depression models; and age, gender and population were covariates in all PLINK analyses. Because of their positive associations (either significantly or as a trend) with both the predictor rs11754661 A allele and the outcome rumination, we could include these two depression phenotypes as covariates (besides age, gender and population) in the linear regression equations described above. For the results see Table 2. Including the two depression phenotypes does not abolish the significant effect of *MTHFD1L* rs11754661 on rumination, but diminishes its effect size (beta) in either an additive or a dominant model (for comparisons also see Table 2). This suggests that depression is only partly responsible for the risk the A allele conveys for rumination.

On the other hand, rumination entirely explains the variance rs11754661 shares with each of the depression phenotypes. Including rumination as an additional predictor in the PLINK regression analyses discussed above, the effect of rs11754661 on depression is no longer statistically significant or a trend in the combined sample: *β*=−0.001; *t*=−0.018; *P*=0.986 in the additive, *β*=0.010; *t*=0.193; *P*=0.847 in the dominant model for BSI depression score, and OR=1.198; *t*=1.189; *P*=0.234 in the additive, OR=1.235; *t*=1.301; *P*=0.193 in the dominant model for lifetime depression.

With regard to the *post hoc* mediation analyses in the Budapest and Manchester samples separately (with age and gender as covariates in all models), in spite of the apparently replicable association with rumination, rs11754661 does not show a significant association with any of the depression phenotypes in Manchester (for BSI depression score, *β*=0.085; *t*=1.088; *P*=0.277 in an additive, and *β*=0.107; *t*=1.252; *P*=0.211 in a dominant model, and for lifetime depression, OR=1.230; *t*=1.276; *P*=0.202 in an additive, and OR=1.289; *t*=1.426; *P*=0.154 in a dominant model). However, we could implement the mediation analyses in the Budapest sample, as rs11754661 associates either significantly or as a trend to both depression phenotypes there (for BSI depression score, *β*=0.141; *t*=1.734; *P*=0.083 in an additive, and *β*=0.151; *t*=1.813; *P*=0.070 in a dominant model, and for lifetime depression, OR=1.775; *t*=2.226; *P*=0.026 in an additive, and OR=1.737; *t*=2.088; *P*=0.037 in a dominant model), and because rumination shows a significant positive association with both depression phenotypes (Pearson *r*=0.536; *P*<0.001 for BSI depression score; and *t*=−9.603; *P*<0.001 for lifetime depression, with a mean rumination score of 1.866±0.017 in those who did not, and of 2.226±0.035 in those who did report lifetime depression). In the mediation analyses in Budapest, we could replicate our findings seen in the combined sample. The two depression phenotypes as predictors diminish but do not abolish the effect of rs11754661 on rumination (*β*=0.094; *t*=2.051; *P*=0.041 in the additive, and *β*=0.090; *t*=1.921; *P*=0.055 in the dominant model; for comparisons see Table 3), whereas rumination as a predictor entirely abolishes the effect of rs11754661 on both depression phenotypes (for BSI depression score, *β*=0.014; *t*=0.210; *P*=0.834 in the additive, and *β*=0.025; *t*=0.361; *P*=0.719 in the dominant model, and for lifetime depression, OR=1.471; *t*=1.425; *P*=0.154 in the additive, and OR=1.443; *t*=1.322; *P*=0.186 in the dominant model).

The discrepancy in the detected effect of rs11754661 on rumination and the non-detected one of rs1801133 cannot be attributed to decreased power. Assuming an *R*^2^=1% and under a dominant model (mean rumination score is 2.13 and its s.d. is 0.58 in case of both SNPs), the power to detect an rs11754661 main effect on rumination (*n*=2120) is 99.6%, whereas the power of rs1801133 (*n*=2204) is 99.7%. In addition, rs1801133 has no effect on either depression phenotypes in the combined sample (BSI depression: *β*=0.004; *t*=0.135; *P*=0.892 in an additive, *β*=0.012; *t*=0.305; *P*=0.761 in a dominant, *β*=−0.010; *t*=−0.177; *P*=0.859 in a recessive model; lifetime depression: OR=1.029; *t*=0.407; *P*=0.684 in an additive, OR=1.047; *t*=0.482; *P*=0.630 in a dominant, OR=1.016; *t*=0.113; *P*=0.910 in a recessive model), suggesting that its lack of effect on ruminative response style is not spurious.

## Discussion

Among polymorphisms of folate pathway genes, the widely investigated *MTHFR* rs1801133 is not associated with ruminative response style in our large combined European white sample, whereas the AD genome-wide marker *MTHFD1L* rs11754661 A allele represents a risk for higher ruminative response style. This association is replicated separately in the Budapest and Manchester cohorts. Moreover, this association is only partly mediated by current depression score and lifetime depression, but ruminative response style fully explains the variance that *MTHFD1L* rs11754661 shares with these depression phenotypes.

That there is an effect of the *MTHFD1L* variant but not of the *MTHFR* is in line with findings of the genome-wide mega-analysis on major depressive disorder by Ripke and colleagues,^39^ where the index SNP of the *MTHFD1L* gene (SNP with the highest significance) showed a more significant association (rs563440; *P*=0.004) with major depression than the index SNP from *MTHFR* (rs17037425; *P*=0.079).

### Discrepancy in the effects of *MTHFR* and *MTHFD1L*

**Figure 2:**
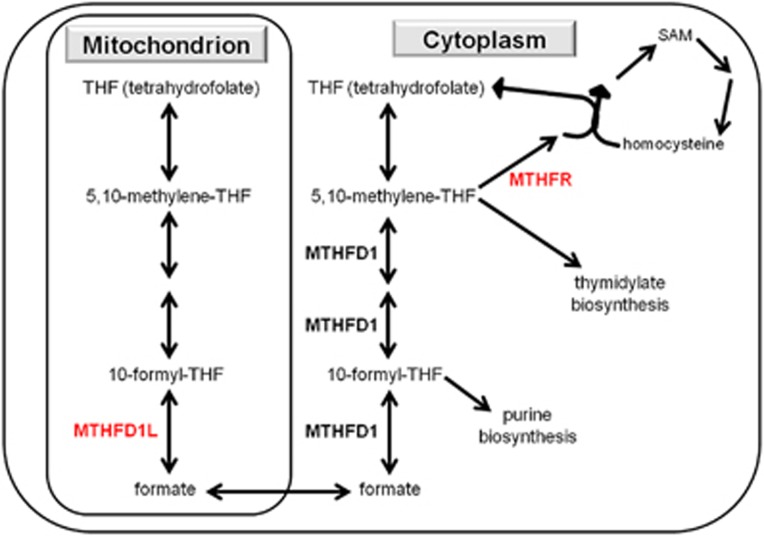
Distinct roles of enzymes MTHFD1L and MTHFR in the folate-related one-carbon cycle. MTHFD1L: mitochondrial monofunctional C_1_-tetrahydrofolate synthase enzyme; MTHFD1: cytoplasmic trifunctional C_1_-tetrahydrofolate synthase enzyme; MTHFR: 5,10-methylenetetrahydrofolate reductase enzyme; SAM: S-adenosylmethionine (a methyl donor in numerous reactions). The arrows represent different reactions or a flow between the mitochondrion and cytoplasm (as appropriate), and the most important enzymes (with bold) and substrates are represented.

There may be several interrelated reasons for the discrepancy in the effects of *MTHFD1L* and *MTHFR*. First, the two enzymes have distinct biochemical roles (see Figure 2 and refs. 29, 40, 41). Specifically, MTHFD1L could enhance both the 10-formyl-THF generation and, by producing formate for the cytoplasm, the synthesis of S-adenosylmethionine (SAM). 10-formyl-THF generation is protective for mitochondria,^22^ whereas SAM is an important methyl donor in epigenetic regulation processes related to memory, learning, cognition and behavior,^18^ and is also crucial in the synthesis of dopamine, serotonin and noradrenaline in the brain.^41^ In contrast, MTHFR can support only one of these two directions, namely 10-formyl-THF generation or SAM synthesis, at the expense of the other one.^22, 42^

A second source of the discrepancy could be the distinct subcellular localization of the two enzymes.^29, 40^ As the protein C_1_-THF synthase coded by *MTHFD1L* is localized in mitochondria, whereas the MTHFR protein coded by *MTHFR* is present in the cytoplasm, we can conclude that the folate pathway in the mitochondria is essential in rumination and other cognitive processes. Furthermore, mitochondrial dysfunction has been associated with depression earlier.^43, 44^

A third reason for the discrepancy could be the distinct sensitivity of these enzymes to other factors, such as environmental effects. For example, it has been demonstrated that the effects of *MTHFR* rs1801133 genotype on the plasma homocysteine level,^30, 45^ DNA methylation level^42^ and cognitive performance^22^ are modulated by the folate status, namely this polymorphism has stronger effect in case of low level of folate compared with high level of folate. In contrast, the *MTHFD1L* gene has pleiotropic effects on the plasma homocysteine level and markers of genome-wide DNA methylation after controlling for nutrient status.^30^ Future research should reveal whether the effect of *MTHFD1L* rs11754661 on ruminative response style depends on the folate status.

### Pathophysiological specificity of ruminative response style?

We found *MTHFD1L* rs11754661 more consistently associated with ruminative response style than with our two depression phenotypes, as rs11754661 does not predict depression in the Manchester sample. Moreover, in Budapest, and in the combined sample, the association of the *MTHFD1L* variant and ruminative response style is only partly mediated by depression, but completely accounts for the effect that the *MTHFD1L* variant exerts on depression. These findings correspond well with two reviews stating that rumination confers a risk not specifically for depression, but, for several psychopathologies, alterations in mental and physical health.^3, 7^ Taken together these observations, the *MTHFD1L* gene and thus the folate pathway may be important in the pathophysiology of other health conditions related to ruminative response style.

Regarding psychiatric disorders, the latest pathway-based genome-wide association studies by the Psychiatric Genomic Consortium^46^ found that methylation pathways, inclusive of the SAM-dependent methyltransferase activity, are among the most important in the common background of major depression, bipolar depression and schizophrenia. Interestingly, the 'one-carbon pool by folate' pathway, which contains the *MTHFR* and *MTHFD1L* genes, was nominally significant for bipolar disorder, showed a trend for major depressive disorder and was not significant for schizophrenia, suggesting that this pathway has distinct effect on mood disorders, probably through a common intermediate phenotype, such as ruminative response style. However, our positive findings with distinctive role of *MTHFR* and *MTHFD1L* polymorphisms still underline the importance of not only the pathway-, but also the gene- or polymorphism-based approach.

Taking into account non-psychiatric disorders, cardiovascular diseases could also be potential targets of future investigations because rumination, denoting a cognitive perseveration on distress, yields a prolonged stress response and slower cardiovascular recovery, and thus a risk for cardiovascular disease.^4, 5^ In addition, cardiovascular diseases share a pattern of alterations in the key one-carbon cycle components (levels of, for example, folate, homocysteine and the universal methyl donor SAM) with psychiatric disorders.^18^

### Therapeutic implications

There is some evidence that methylfolate^47^ and SAM^41^ supplementations are effective in the treatment of major depression; however, the evidence that folate augments the efficacy of conventional antidepressant medication is mixed and includes a recent large negative study.^48, 49^ Our present results and previous genetic association studies may shed light on these contradictory findings. First, not the entire folate pathway is associated with depression^39, 46^ and treatment response,^49^ but elements with stronger influence on methylation processes^30^ have more consistent effects. Alterations in the DNA and histone methylations, which translate environmental exposures to specific gene expression patterns and are major factors in the regulation of brain development and synaptic plasticity, may cause long-term increased risk for depression,^50^ which is difficult to reverse by supplementation therapy. Second, ruminative response style, a trait-like risk factor for several psychiatric and physical disorders, represents an intermediate phenotype between *MTHFD1L* polymorphism and depression. Thus, methylfolate and SAM supplementations may be more effective in those with high ruminative response style, as an augmentation of targeted psychotherapies,^51^ although this hypothesis has not been tested yet.

### Limitations

Our study is cross-sectional and cannot address the time course of an association between *MTHFD1L* rs11754661 and either ruminative response style or depression. In addition, it cannot account directly for reporting bias for past depressive episodes; however, we measured depression in two ways, one of which is current depression, allowing some confidence that reporting bias does not explain the association. Moreover, determining the precedence of rumination or lifetime depression episodes would be of crucial importance in our study, as we operationalized rumination specifically as ruminative response style, anchoring it to an answer to sadness or depressed mood. However, narrowing the concept of rumination like this makes it easier to interpret our findings. Our lifetime depression measure was not based on face-to-face diagnostic interviews, but had been validated in a subsample. In addition, further research covering the whole genes with haplotype tags or sequencing these regions is required to confirm the findings about the effects of single SNPs.

### Conclusions and implications for future research

In conclusion, we have identified the *MTHFD1L* rs11754661 A allele as a genetic risk factor for ruminative response style, and this association may convey pathophysiological implications for not only depression but also other mental and physical disorders. This association, which replicated in two independent European white samples, enriches our knowledge about the genetic architecture of ruminative response style. In addition, the folate pathway can be linked to most of the previously described genetic risk factors for rumination. Therefore, future research is needed to shed light on the particular ways in which *MTHFD1L* rs11754661 might affect rumination, for example, via homocysteine levels, synaptic plasticity, methylation patterns of relevant genes and methylation-related dynamics of monoamine metabolism. It will also be crucial to determine whether *MTHFD1L* acts at specific points in neural development when the tendency to ruminate is established.

## Figures and Tables

**Table 1:** Description of the population samples

	*Budapest*	*Manchester*	*Budapest+Manchester*	*Difference between Budapest and Manchester*
*Gender*				
Female (%)	624 (69.7%)	916 (70%)	1540 (69.9%)	*X*^2^=0.017; *P*=0.897
Male (%)	271 (30.3%)	393 (30%)	664 (30.1%)	
				
Age (mean±s.e.m.)	31.26 (0.355)	34.04 (0.284)	32.91 (0.224)	*t*=−6.153; *P*<0.001

*MTHFR rs1801133*				
TT (%)	122 (13.6%)	154 (11.8%)	276 (12.5%)	*X*^2^=1.717; *P*=0.424
TC (%)	400 (44.7%)	602 (46%)	1002 (45.5%)	
CC (%)	373 (41.7%)	553 (42.2%)	926 (42%)	

*MTHFD1L rs11754661*				
AA (%)	1 (0.1%)	10 (0.8%)	11 (0.5%)	*X*^2^=9.914; *P*=0.007
GA (%)	75 (8.7%)	148 (11.8%)	223 (10.5%)	
GG (%)	786 (91.2%)	1100 (87.4%)	1886 (89%)	
				
Rumination score (mean±s.e.m.)	1.94 (0.016)	2.25 (0.017)	2.13 (0.012)	*t*=−13.104; *P*<0.001	
BSI depression score (mean±s.e.m.)	0.56 (0.023)	1.07 (0.028)	0.86 (0.020)	*t*=−13.954; *P*<0.001	
	
*Lifetime depression*					
Reported (%)	192 (21.5%)	734 (56.1%)	926 (42%)	*X*^2^=261.521; *P*<0.001	
Not reported (%)	703 (78.5%)	575 (43.9%)	1278 (58%)		

Abbreviations: BSI, Brief Symptom Inventory; *MTHFR*, 5,10-methylenetetrahydrofolate reductase.

The Manchester sample shows significantly higher mean age, rumination score and BSI depression score, and higher frequencies of reported lifetime depression and of the *MTHFD1L* rs11754661 A allele than the Budapest sample.

**Table 2:** Linear regression models for rumination score as an outcome variable, separately with the two SNPs

	*MTHFR rs1801133*						*MTHFD1L rs11754661*											
	*Predictor variable*	N	*Beta*	*s.e.*	t	P	*Predictor variable*	*N*	*Beta*	*s.e.*	t	P	*Predictor variable*	N	*Beta*	*s.e.*	t	P
Additive	*MTHFR* rs1801133	2204	−0.023	0.017	−1.358	0.175	*MTHFD1L* rs11754661	2120	**0.112**	**0.035**	**3.182**	**0.001**	*MTHFD1L* rs11754661	2117	**0.070**	**0.029**	**2.388**	**0.017**
	Age	2204	−0.006	0.001	−5.296	<0.001	Age	2120	−0.006	0.001	−5.214	<0.001	Age	2117	−0.006	0.001	−6.347	<0.001
	Gender	2204	0.273	0.025	10.710	<0.001	Gender	2120	0.274	0.026	10.590	<0.001	Gender	2117	0.177	0.022	8.161	<0.001
	Population	2204	0.321	0.024	13.440	<0.001	Population	2120	0.321	0.024	13.210	<0.001	Population	2117	0.103	0.022	4.795	<0.001
													BSI depression score	2117	0.284	0.012	23.570	<0.001
													Lifetime depression	2117	0.217	0.024	9.199	<0.001
																		
Dominant	*MTHFR* rs1801133	2204	−0.043	0.024	−1.825	0.068	*MTHFD1L* rs11754661	2120	**0.122**	**0.038**	**3.228**	**0.001**	*MTHFD1L* rs11754661	2117	**0.072**	**0.031**	**2.309**	**0.021**
	Age	2204	−0.006	0.001	−5.317	<0.001	Age	2120	−0.006	0.001	−5.213	<0.001	Age	2117	−0.006	0.001	-6.346	<0.001
	Gender	2204	0.273	0.025	10.710	<0.001	Gender	2120	0.274	0.026	10.570	<0.001	Gender	2117	0.177	0.022	8.146	<0.001
	Population	2204	0.321	0.024	13.460	<0.001	Population	2120	0.322	0.024	13.230	<0.001	Population	2117	0.104	0.022	4.815	<0.001
													BSI depression score	2117	0.284	0.012	23.560	<0.001
													Lifetime depression	2117	0.217	0.024	9.201	<0.001
																		
Recessive	*MTHFR* rs1801133	2204	−0.002	0.035	−0.058	0.954												
	Age	2204	−0.006	0.001	−5.277	<0.001												
	Gender	2204	0.273	0.025	10.700	<0.001												
	Population	2204	0.321	0.024	13.450	<0.001												

Abbreviations: BSI, Brief Symptom Inventory; *MTHFR*, 5,10-methylenetetrahydrofolate reductase; SNP, single-nucleotide polymorphism.

PLINK linear regression equations were constructed with the predictor variables displayed in the rows. Additive, dominant and recessive models were run in the combined sample, separately with both SNPs as predictors. T is the minor allele in case of *MTHFR* rs1801133; A in case of *MTHFD1L* rs11754661. Recessive models have not been run for rs11754661 because of low number in the AA group. Significance threshold is *P⩽*0.010 (Bonferroni-corrected) for the main analyses, and *P⩽*0.050 for *post hoc* analyses (those including also the two depression phenotypes as covariates). Significant findings for the SNPs as a predictor variable are marked with bold.

**Table 3:** Linear regression models of *MTHFD1L* rs11754661 for rumination score as an outcome variable, separately in Budapest and Manchester

	*Budapest*						*Manchester*					
	*Predictor variable*	N	*Beta*	*s.e.*	t	P	*Predictor variable*	N	*Beta*	*s.e.*	t	P
Additive	*MTHFD1L* rs11754661	862	**0.158**	**0.054**	**2.915**	**0.004**	*MTHFD1L* rs11754661	1258	**0.095**	**0.046**	**2.049**	**0.041**
	Age	862	*−*0.004	0.002	*−*2.417	0.016	Age	1258	*−*0.008	0.002	*−*4.693	<0.001
	Gender	862	0.220	0.034	6.409	<0.001	Gender	1258	0.311	0.037	8.456	<0.001
												
Dominant	*MTHFD1L* rs11754661	862	**0.157**	**0.055**	**2.828**	**0.005**	*MTHFD1L* rs11754661	1258	**0.107**	**0.050**	**2.120**	**0.034**
	Age	862	*−*0.004	0.002	*−*2.395	0.017	Age	1258	*−*0.008	0.002	*−*4.700	<0.001
	Gender	862	0.220	0.034	6.419	<0.001	Gender	1258	0.310	0.037	8.437	<0.001

PLINK linear regression equations were constructed with the predictor variables displayed in the rows. Additive and dominant models were run, separately in Budapest and Manchester; all with A as the minor allele. (Recessive models have not been run because of low number in AA groups.) Significant findings for *MTHFD1L* rs11754661 as a predictor variable are marked with bold.

## Notes

JFWD has variously performed consultancy, speaking engagements and research for Bristol-Myers Squibb, AstraZeneca, Eli Lilly, Schering Plough, Janssen-Cilag and Servier (all fees are paid to the University of Manchester to reimburse them for the time taken); he also has share options in P1vital. IMA has received consultancy fees from Servier, Alkermes, Lundbeck/Otsuka and Janssen, an honorarium for speaking from Lundbeck and grant support from Servier and AstraZeneca. RE has received consultancy fees from Cambridge Cognition and P1vital. The remaining authors declare no conflict of interest.
